# MYBL2 is a Novel Independent Prognostic Biomarker and Correlated with TMB in pancreatic cancer

**DOI:** 10.7150/jca.96320

**Published:** 2024-06-11

**Authors:** Yanping Li, Shanshan Wang, Miao Guo, Rui Yang, Xiaonan Wei, Haibin Li, Siyuan Yan

**Affiliations:** 1 Precision Medicine Laboratory for Chronic Non-communicable Diseases of Shandong Province, Institute of Precision Medicine, Jining Medical University, Jining, Shandong 272067, China.; 2 College of life science,Shandong First Medical University, Jinan, Shandong 250000, China.; 3 Biomedical Laboratory, Medical School of Liaocheng University, Liaocheng, Shandong 252000, China.

**Keywords:** MYBL2, pancreatic cancer, prognosis, survival analysis, TMB

## Abstract

**Background:** Pancreatic cancer continues to pose a significant threat due to its high mortality rate. While MYB family genes have been identified as oncogenes in certain cancer types, their role in pancreatic cancer remains largely unexplored.

**Methods:** The mRNA and protein expression of MYB family genes in pancreatic cancer samples was analyzed using TNMplot, HPA, and TISBID online bioinformatics tools, sourced from the TCGA and GETx databases. The relationship between MYB family gene expression and survival time was assessed through Kaplan-Meier analysis, while the prognostic impact of MYB family gene expression was evaluated using the Cox proportional hazards model. Additionally, Spearman's correlation analysis was employed to investigate the correlation between MYB family genes and TMB/MSI.

**Results:** The integration of data from various databases demonstrated that all MYB family genes exhibited dysregulated expression in pancreatic cancer. However, only the expression of the MYBL2 gene displayed a notable association with the grade and stage of pancreatic cancer. Furthermore, the MYBL2 gene exhibited significant variations in both univariate and multivariate factor analyses.Subsequent functional analyses revealed a significant correlation between MYBL2 expression in pancreatic cancers and various biological processes, such as DNA replication, tumor proliferation, G2M checkpoint regulation, pyrimidine metabolism, and the P53 pathway. Additionally, a notable positive association was observed between MYBL2 expression and tumor mutational burden (TMB), a predictive indicator for response to PD1 antibody treatment.

**Conclusion:** MYBL2 may be a double marker for independent diagnosis and PD1 antibody response prediction of pancreatic cancer patients

## Introduction

Pancreatic cancer is a highly aggressive neoplasm with a significant mortality rate that predominantly impacts the gastrointestinal system on a global scale 1. According to the latest data, cancer is the sixth leading cause of cancer death in China 2, The outlook for individuals suffering from this condition is extremely unfavorable, as indicated by a survival rate of only 5% over a period of five years 3. Pancreatic cancer exhibits notable heterogeneity, with molecular targeted therapies demonstrating restricted effectiveness in combating the disease 4. Furthermore, in the majority of instances, pancreatic cancer is diagnosed at a late stage, often after it has progressed locally or spread to distant organs, due to the lack of reliable early detection methods 5. Early prognosis and risk assessment for patients with pancreatic cancer is crucial in clinical practice. Therefore, there is an urgent need to study the genes linked to the onset, progression, and prognosis of pancreatic cancer in order to establish a theoretical basis for early detection and personalized treatment.

The MYB family of transcription factors exhibit a broad distribution across eukaryotic organisms and play diverse roles in biological processes such as cancer progression, immune dysfunction, and developmental abnormalities 6. Similar to humans, the MYB gene family is comprised of three members: MYB, MYBL1, and MYBL2, which are responsible for encoding the transcription factors c-MYB, A-MYB, and B-MYB 7. Research has demonstrated that MYB family members exhibit abnormal expression in various human cancers, indicating their potential involvement in tumorigenesis and disease progression 8. MYB is identified in hematopoietic cells, colonic crypts, and the brain of mammals, while MYBL1 is observed in the developing central nervous system, germinal B-lymphocytes, and reproductive systems 9. Studies have identified MYB as a novel regulator of pancreatic tumor desmoplasia, indicating its potential multifaceted roles in the pathobiology of pancreatic cancer 10. The aberrant overexpression of MYB in pancreatic cancer cells serves as a critical factor in determining both tumor growth and metastatic potential 11. A study revealed that 80% of colorectal cancers demonstrate heightened MYB levels, leading to the formation of aggressive tumors and a less favorable prognosis 12. Xie *et al.* have previously shown that MYBL1 enhances the proliferation and metastasis of hepatocellular carcinoma (HCC) cells by transcriptionally upregulating TWIST1 expression 13. MYBL2 is ubiquitously expressed in proliferating cells and plays a crucial role in regulating cell cycle progression, survival, and differentiation 14, 15. Studies have demonstrated that MYBL2 functions as an oncogene and influences the prognoses of breast, liver, and glioma cancers, as well as the response to immunotherapy 16-19. The clinical relevance and molecular pathways associated with MYB family genes in relation to prognosis among pancreatic cancer patients are not yet fully understood. The utilization of bioinformatics tools has facilitated a more comprehensive comprehension of molecular mechanisms in disease states. In this study, we examined the impact of MYB family gene expression on prognosis in pancreatic cancer and conducted various bioinformatics analyses to investigate the associated cell functions and pathways. Additionally, we explored the potential correlation between MYB, MYBL1, and MYBL2 with tumor mutational burden (TMB) and microsatellite instability (MSI). The results of this research offer additional understanding of the potential molecular mechanisms of these genes in pancreatic cancer and their impact on a patient's clinical prognosis.

## Materials and Methods

### Differential expressions data source and Analysis

The expression distribution of MYB family gene which including three members: MYB, MYBL1and MYBL2. RNA-sequencing expression (level 3) profiles for MYB, MYBL1 and MYBL2 from 179 tumor tissues were downloaded from the TCGA dataset (https://portal.gdc.com) and 332 normal tissues including 4 paracancer samples from the TCGA database and 328 samples collected from 54 undiseased tissue sites of nearly 1000 individuals from the GETx database (https://commonfund.nih.gov/GTEx/).To verify the differential MYB, MYBL1 and MYBL2 expressions in pancreatic tumor and normal tissues, we also used TNMplot, which is a public free database developed by department of Bioinformatics of the Semmelweis University (https://tnmplot.com/analysis/). Statistical analyses were performed using R software v4.0.3 (R Foundation for Statistical Computing, Vienna, Austria). *p < 0.05, **p < 0.01, ***p < 0.001, asterisks (*) stand for significance levels. The statistical difference of two groups was compared through the Wilcox test. The protein expression level of MYB, MYBL1and MYBL2 in PAAD cancers were identified by the Human Protein Atlas (HPA) database (https://www.proteinatlas.org/).We also used the TISBID website (http://cis.hku.hk/ TISIDB/index.php) to investigate the association between the expression of MYB, MYBL1, MYBL2 and the stage and grade of PAAD cancers. The expression data are first log2(TPM+1) transformed for differential analysis. The method for differential gene expression analysis is one-way ANOVA, using pathological stage as variable for calculating differential expression.

### Clinical bioinformatics analysis

A clinical bioinformatics database (www.aclbi.com) is a multifunctional analytical platform. The RNA-sequencing expression profiles (level 3) of the MYB family in pancreatic cancer patients, along with their corresponding clinical information, were obtained from The Cancer Genome Atlas (TCGA) dataset (https://portal.gdc.com). This data was utilized to investigate the association between mRNA expression of the MYB family and various clinicopathological parameters of pancreatic cancer including vital status, age, sex, race, new tumor event type, smoking, radiation therapy, history of neoadjuvant treatment and therapy type. The TISBID website (http://cis.hku.hk/TISIDB/index.php) was utilized for the purpose of examining the correlation between MYBL2 expression and the stage and grade of pancreatic cancers. The measurement data are displayed as the mean ± SD. Unpaired t-test was used for analyzing statistical assessments. The association between MYB gene family and clinical characteristic variables was analyzed using Pearson chi-squared test.

### Identification of the Prognostic Factors for OS, DSS and PFS in PAAD

Univariate and multivariate Cox regression analyses were employed to evaluate the influence of MYB, MYBL1, MYBL2, as well as five significant clinical and prognostic factors (age, gender, race, grade, pTNM-stage) on prognosis from the TCGA dataset (https://portal.gdc.com). The utilization of the forestplot R package facilitated the presentation of the P value, HR, and 95% CI for each variable in the forest. Subsequently, a nomogram was constructed using the outcomes of the multivariate cox proportional hazards analysis to forecast the overall recurrence within 1, 3 and 5 years. The nomogram visually depicted the factors contributing to the risk of recurrence for an individual patient, with the points assigned to each risk factor calculated through the rms R package. An analysis of Kaplan-Meier survival data from the TCGA dataset was performed using Log-rank test to compare differences in survival curves, such as overall survival (OS), disease-free survival (DFS) and progression-free survival (PFS) curve among different groups. MYBL2 mRNA predictive accuracy was compared using time ROC analysis (v0.4). An analysis method and R package were implemented using R (foundation for statistical computing 2020) version 4.0.3. P values less than 0.05 were considered statistically significant.

### Distribution of MYB family gene expression in different cell lines

To observe the expression of MYB family genes in pancreatic cancer in order to obtain the expression of this family of genes in different cell lines as a way to select suitable cell lines for subsequent analysis or validation. Cell line mRNA expression matrices for tumors were obtained from the CCLE dataset (https://portals.broadinstitute.org/ccle).The analysis was constructed by the R v4.0.3 software package ggplot2 (v3.3.3).

### Correlation analysis of MYBL2 gene and Pathways

In order to gain a better understanding of the molecular mechanisms underlying MYBL2 expression in pancreatic cancer. We obtained RNAseq data and corresponding clinical information for pancreatic tumors from The Cancer Genome Atlas (TCGA) database (https://portal.gdc.com). First we analyzed the differential genes in the two groups of samples with high and low MYBL2 expression and looked at the functions to which these up- or down-regulated genes were enriched by and through functional enrichment. In the enrichment result, p < 0.05 or FDR <0.05 is considered to be a meaningful pathway (enrichment score with -log10 (P) of more than 1.3). R software GSVA package was used to analyze the gene-pathway relationship between MYBL2 and the pathways, the pathway score was analysed with Spearman. p < 0.05 was considered statistically significant.

### TMB and MSI analysis

Biomarkers of immune checkpoint inhibitor response include TMB and MSI. RNA-sequencing expression profiles and corresponding clinical information for pancreatic cancer were downloaded from the TCGA dataset (https://portal.gdc.com). Spearman's correlation analysis to describe the correlation between MYB family and TMB/MSI. P values less than 0.05 were considered statistically significant (*P < 0.05).

## Results

### MYB and MYBL2 expression are high in Pancreatic cancer

**Figure 1:**
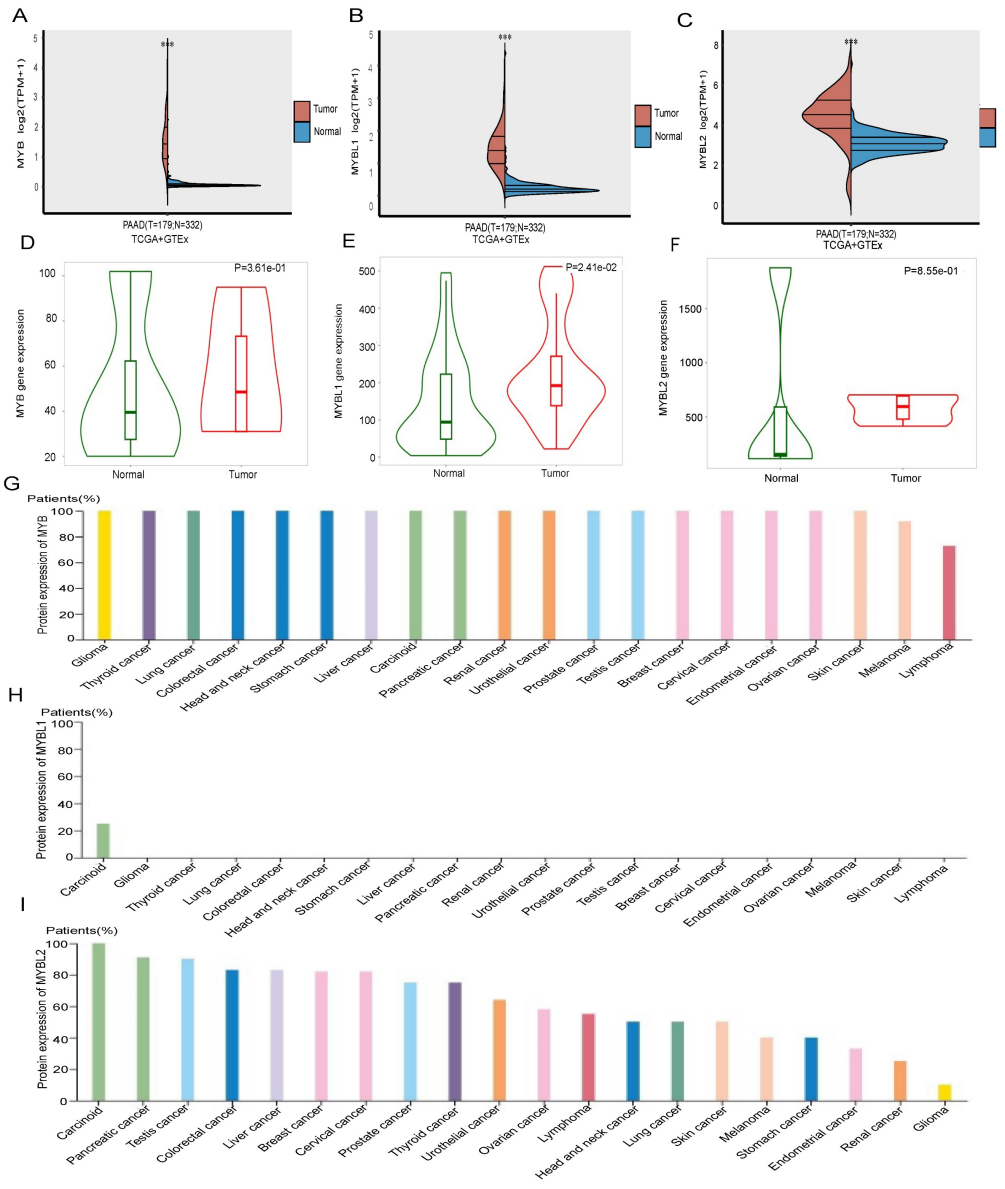
The different mRNA expressions of MYB family members in PAAD and normal tissues. (A) mRNA expressions of MYB in PAAD and normal tissues from TCGA and GETx database. (B) An analysis of the expression of MYB in PAAD tissues and normal tissues using RNA-Seq based data by the TNMplot. (C) mRNA expressions of MYBL1 in PAAD and normal tissues from TCGA and GETx database. (D) An analysis of the expression of MYBL1 in PAAD tissues and normal tissues using RNA-Seq based data by the TNMplot. (E) mRNA expressions of MYBL2 in PAAD and normal tissues from TCGA and GETx database. (F) An analysis of the expression of MYBL2 in PAAD tissues and normal tissues using RNA-Seq based data by the TNMplot. (G)The protein expression level of MYB for each cancer types from the Human Protein Atlas. (H)The protein expression level of MYBL1 for each cancer types from the Human Protein Atlas. (I)The protein expression level of MYBL2 for each cancer types from the Human Protein Atlas.

The expression of MYB family member in pancreatic adenocarcinoma (PAAD) and normal tissues was assessed by analyzing RNA sequencing data obtained from the Cancer Genome Atlas (TCGA) using the R programming language. A total of 172 tumor samples were obtained from the TCGA database, specifically focusing on mRNA expression. Additionally, 332 normal tissues were included in the analysis, comprising of 4 paracancer samples sourced from the TCGA database, as well as 328 samples collected from 54 undiseased tissue sites of approximately 1000 individuals from the GETx database. In the present study, it was observed that the genes MYB, MYBL1, and MYBL2 displayed significantly elevated expression levels in pancreatic adenocarcinoma (PAAD) tumor tissues compared to normal tissues (p < 0.001). (p < 0.001) (Figure 1A-C). The expression of MYB, MYBL1, and MYBL2 in normal tissues and cancers were corroborated through TNMplot platform, thereby confirming their elevated levels in comparison to normal tissues. (Figure 1D-F).

Next, we analyzed the expression of MYB, MYBL1 and MYBL2 at the protein level in pancreatic cancer tissues by HPA database. The findings indicated a high expression of MYB in the majority of cancer tissues, particularly in pancreatic cancer (Figure 1G), while MYBL1 exhibited weak protein expression exclusively in endometrial cancer and was absent in all other cancer tissues (Figure 1H). Additionally, MYBL2 demonstrated the second highest level of expression in pancreatic cancer (Figure 1I). Taken together, these results suggest that MYBL2 may have a more important role in pancreatic cancer than MYB and MYBL1.

### Expression of MYB gene family and clinicopathological parameters in pancreatic cancer patients

**Figure 2:**
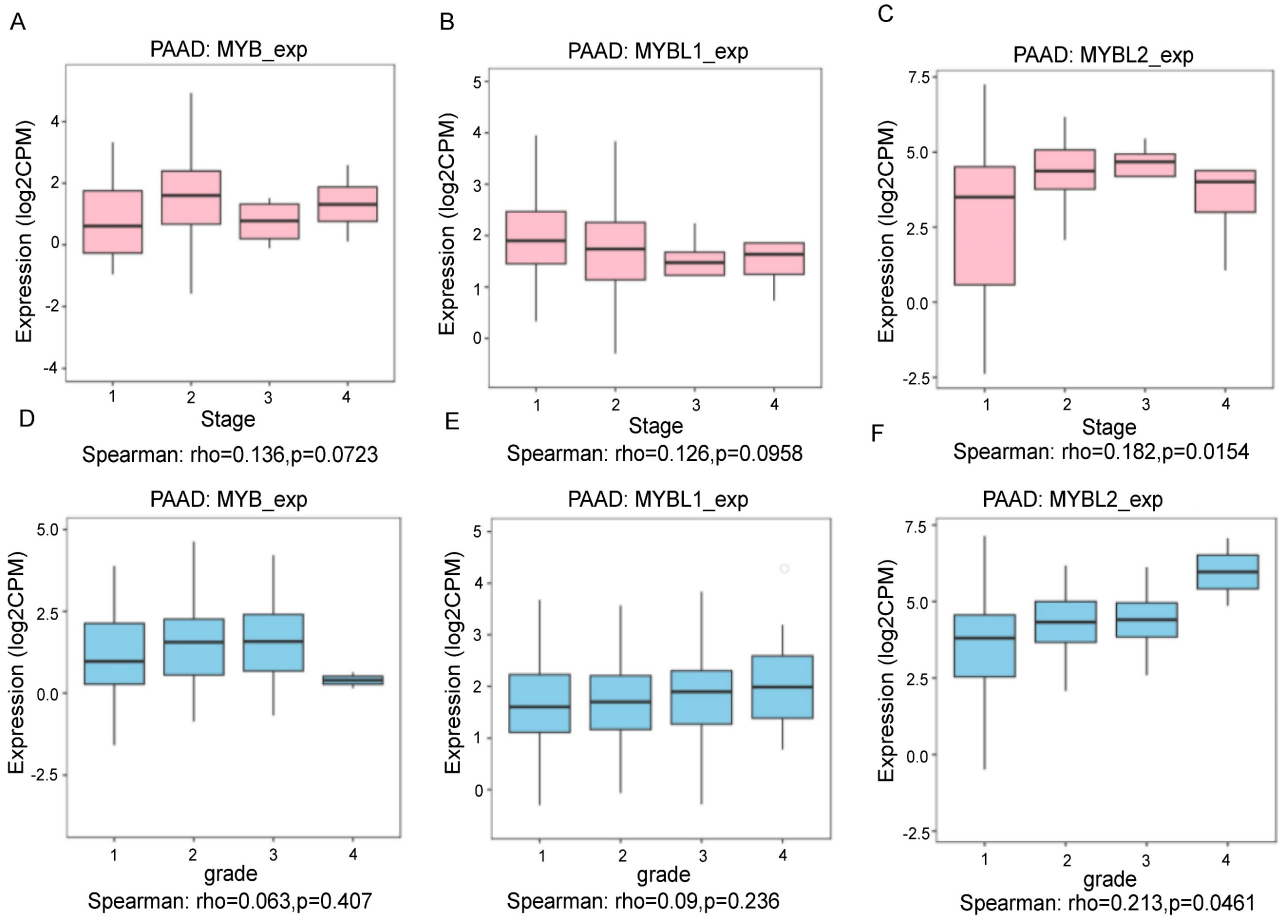
The correlation between MYB family genes expression and the stage and grade of PAAD cancer from the TCGA database analyzed by TISBID tool. The expression levels of MYB(A) MYBL1(B) and MYBL2 (C) based on PAAD cancer stage. The expression levels of MYB (D) MYBL1 (E) and MYBL2 (F) based on PAAD cancer grade.

To evaluate the correlation between the expression of the MYB gene family and the clinical-pathological features of pancreatic cancer by the Clinical Bioinformatics Database (www.aclbi.com). The results showed that the high expression of MYB gene had no significant associations with vital status, age, sex, race, new tumor event type, smoking, radiation therapy, history of neoadjuvant treatment and therapy type compared to low expression group (Table 1). The elevated expression of the MYBL1 gene showed a significant correlation with race (p = 0.027), whereas no significant correlations were observed with vital status, age, sex, new tumor event type, smoking, radiation therapy, history of neoadjuvant treatment, and therapy type when compared to the low expression group (Table 2). The increased expression of the MYBL2 gene exhibited a notable association with vital status (p = 0.009), while no significant associations were found with age, sex, race, new tumor event type, smoking, radiation therapy, history of neoadjuvant treatment, and therapy type in comparison to the low expression group (Table 3). We also conducted an analysis of the relationship between MYB gene family expression and the stages and grades of pancreatic cancer using the TISDIB tool. The findings indicate that the expression levels of MYB and MYBL1 were not significantly correlated with the stage (Figure 2A and B, p=0.0723, p=0.0958), while only MYBL2 expression demonstrated a positive correlation with the stage (Figure 2C, p=0.0154). Furthermore, an analysis of the relationship between the MYB gene family and grade revealed that MYB and MYBL1 expression levels were not associated with grade (Figure 2D and E, p=0.407, p=0.236), with only MYBL2 showing a positive correlation with grade (Figure 2F, p=0.0461).

In addition, we have re-established the relationship between MYB family genes and tumor stage and grade by GEPIA and UALCAN online software. The results of the analysis were consistent with those of the TISDIB analysis, both of which indicated that only the expression of MYBL2 was positively correlated with the stage (Figure S1A-C) and increased with the increase in pancreatic cancer grade (Figure S1D-F).

**Figure 3:**
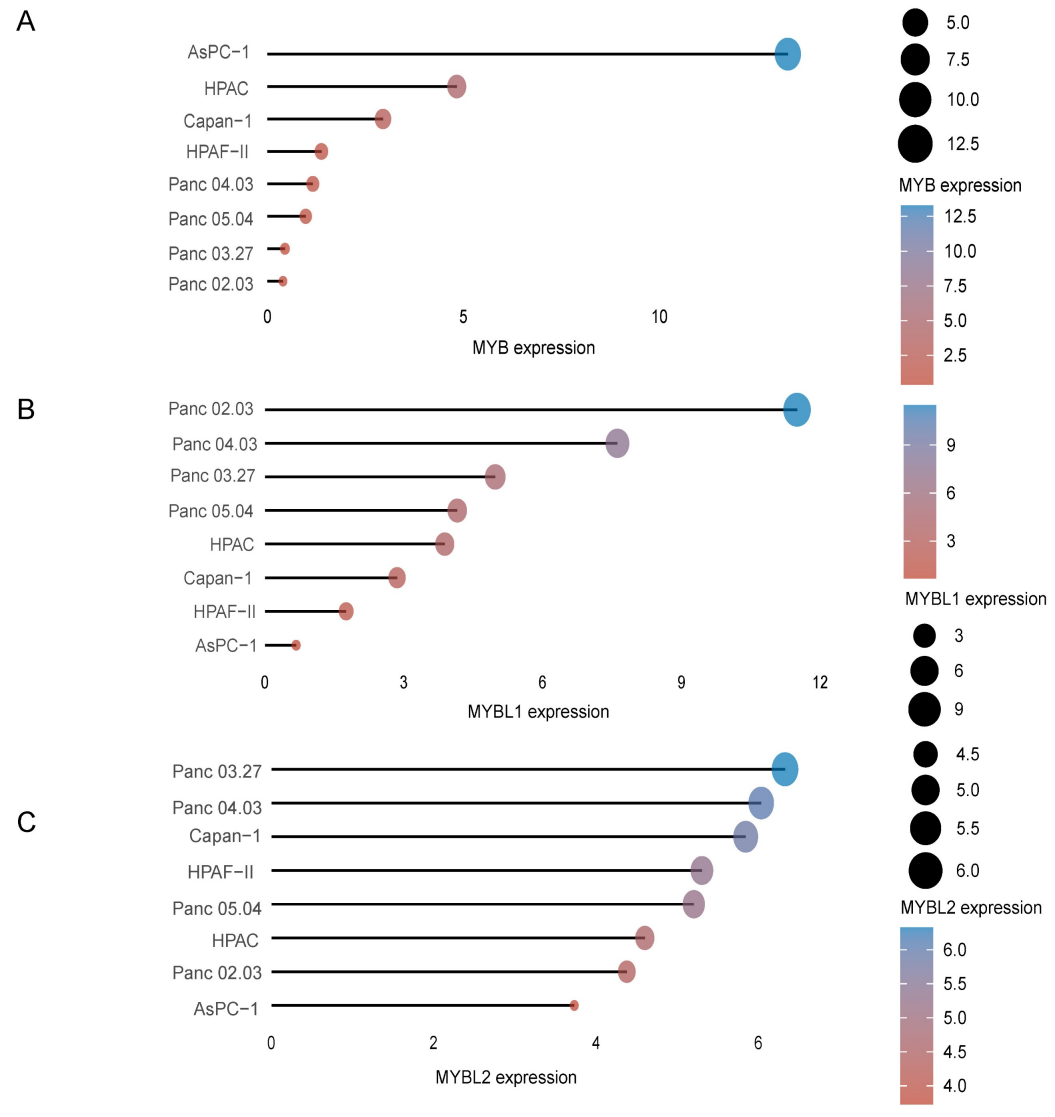
The expression distribution of MYB, MYBL1 and MYBL2 mRNA in different cell lines. The abscissa represents the expression distribution of mRNA and the ordinate represents different cell lines, different colors and the size of dots represent expression.

Moreover, in order to select suitable cell lines for subsequent analysis in pancreatic cancer, we analyzed the expression of MYB family genes in different cell lines of pancreatic cancer from the CCLE dataset (https://portals.broadinstitute.org/ccle). The results of the analysis show that the three MYB family genes are differentially expressed in eight commonly used pancreatic cancer cell lines. MYB expression was highest in the AsPC-1 cell line (Figure 3A), MYBL1 expression was highest in the Panc 02.03 cell line (Figure 3B) and MYBL2 was highest in the Panc 03.27 cell line (Figure 3C), respectively.

### High Expression of MYBL2 is an independent risk factor for PAAD

**Figure 4:**
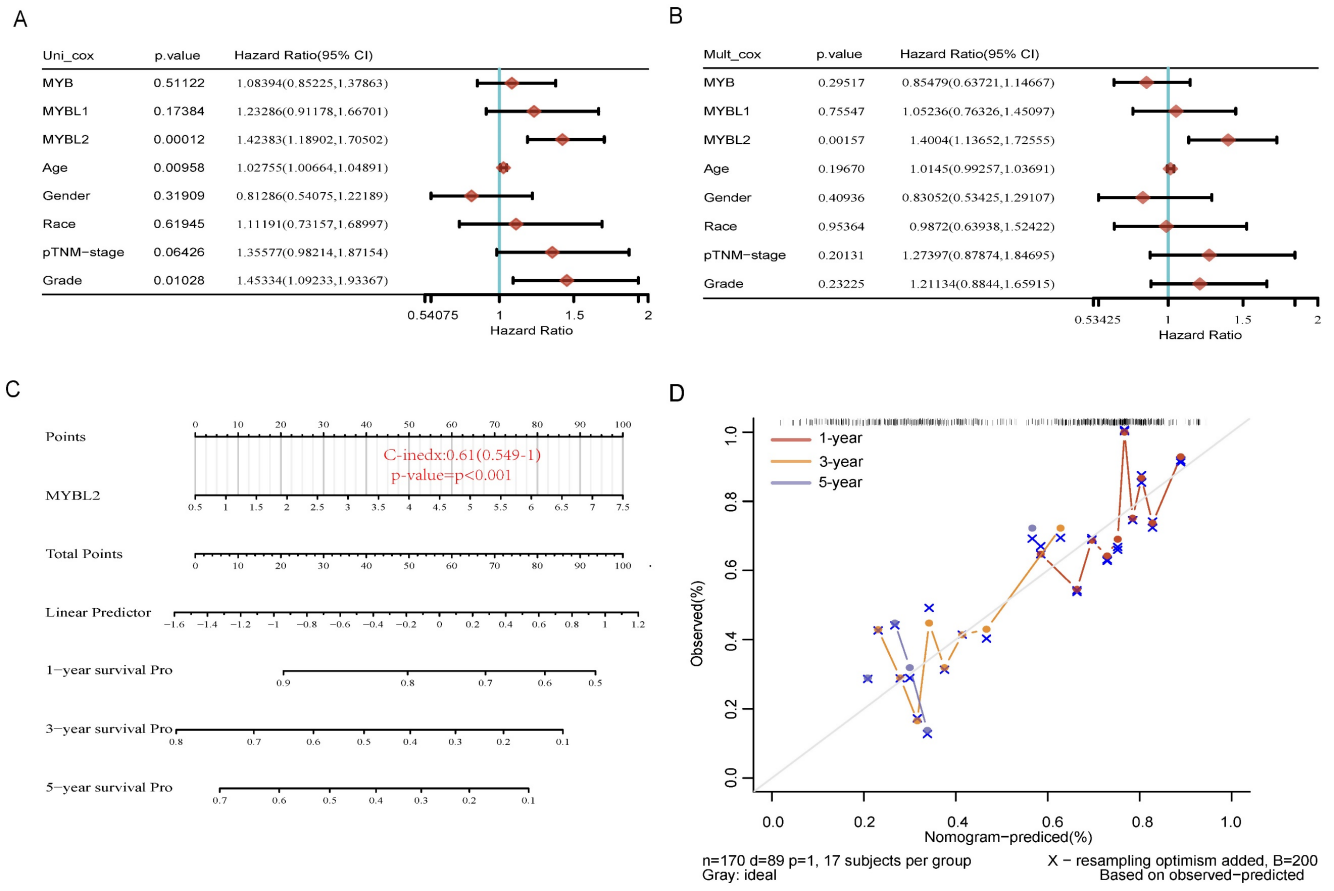
Analyzing the impact of MYB, MYBL1 and MYBL2 genes and clinical factors on overall survival in pancreatic adenocarcinoma (PAAD). Univariate (A) and multivariate Cox regression(B) are used to examine the p-value, risk coefficient (HR), and confidence interval. (C) Nomogram consisting of risk score and other clinical indicators to predict the 1-yr, 3-yr and 5-yr OS of the patients with PAAD. (D) Blue, red, and orange lines represent the 1-year, 3-year, and 5-year observed nomograms, respectively, while the dashed lines represent the calibration curve for the discovery group's overall survival nomogram.

To investigate the relationship between MYB family gene expression and survival and prognosis of pancreatic cancer patients. We performed single and multiple regression analyses of MYB family genes by the Clinical Biosignal House platform (www.aclbi.com). The univariable analysis results presented in Figure 4A indicate that MYB (HR=1.08394, p=0.51122) and MYBL1 (HR=1.23286, p=0.17384) are not significantly associated with prognosis. However, MYBL2 (HR=1.42383, p=0.00012), age (HR=1.02755, p=0.00958), and grade (HR=1.45334, p=0.01028) were found to have a significant impact on pancreatic cancer prognosis. The subsequent multivariable regression analysis revealed that significance only for MYBL2 expression (Figure 4B, HR=1.4004, p=0.00157). These results demonstrated that MYBL2 expression was an independent prognostic factor in PAAD. Subsequently, in order to provide some guidance for clinical prognosis, based on the results of multifactorial cox regression analysis, a column-line diagram of variables with significant differences in prognosis (nomogram) was constructed. MYBL2 was visualized in the nomogram. Nomograms of 1-yr, 3-yr, and 5-yr OS in the cohort are shown in Figure 4C. The nomogram model is close to the calibration curve, indicating that the model predicts well (Figure 4D).

**Figure 5:**
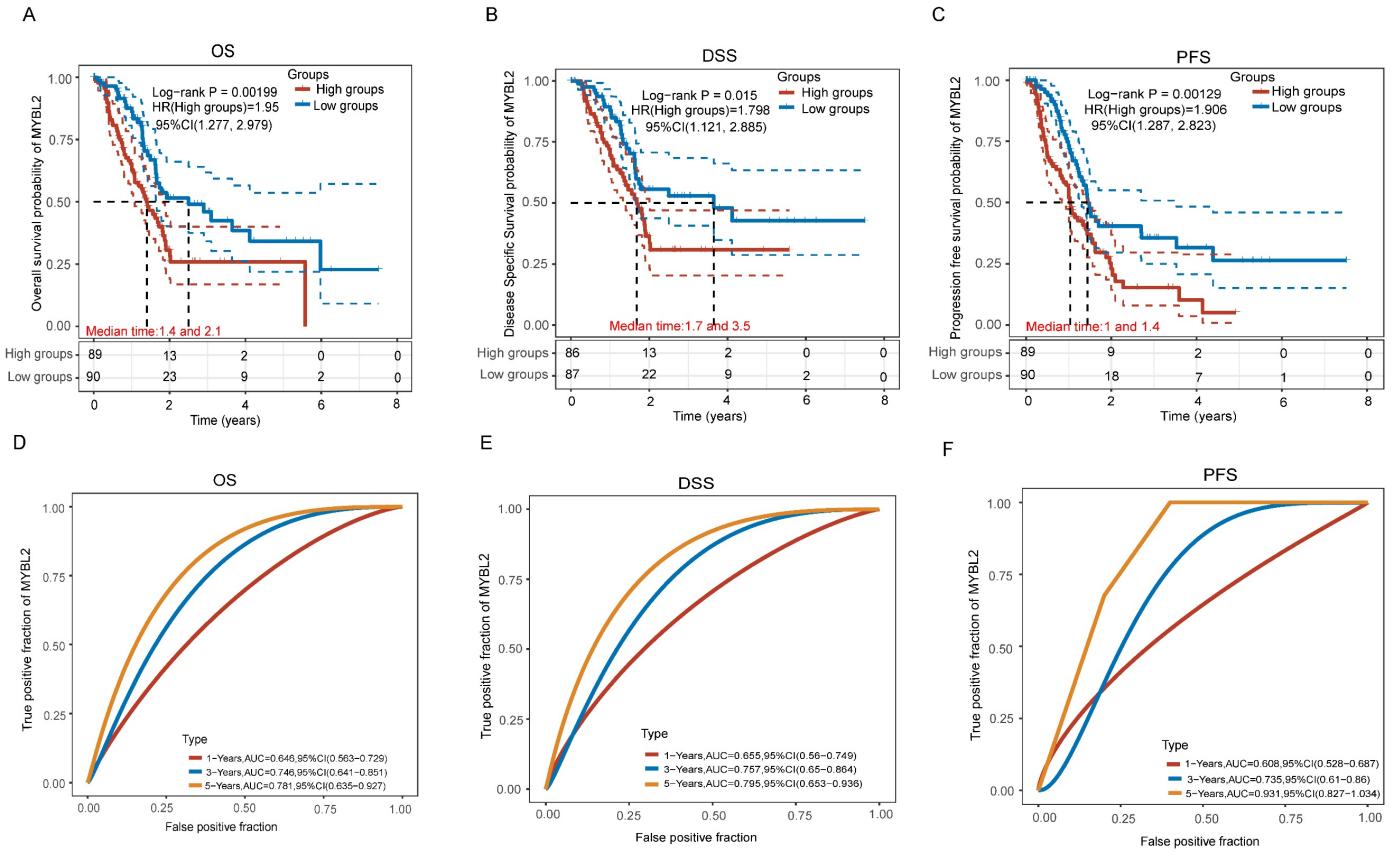
The correlation between MYBL2 expression and OS (A), DSS (B) and PFS (C) from TCGA dataset, comparison among different groups was made by log-rank test. The predictive accuracy of MYBL2 mRNA at different times was analyzed using ROC curves and AUC values (D, E, F).

To further determine the association of the MYB family with pancreatic cancer survival, we analyzed the KM survival curves including OS, DSS and PFS for the MYB family genes in the TCGA data. In individuals with PAAD, high MYBL2 expression was significantly associated with overall survival time (Figure 5A, p=0.00199), Disease Free Survival time (Figure 5B, p=0.015) and Progression Free Survival time (Figure 5C, p=0.00129). Furthermore, the risk coefficients HR were all greater than 1, indicating that the higher the expression of MYBL2, the worse the prognosis of pancreatic cancer patients. We also analyzed the ROC curves and AUC values of the MYBL2 gene at cancer at 1-year, 3-year and 5-year. The analysis showed that the AUC values of the prognostic models of OS (Figure 5D), DSS (Figure 5E) and PFS (Figure 5F) were all greater than 7.0 or above, indicating that the MYBL2 gene is highly accurate in predicting survival in pancreatic cancer patients. Furthermore, the OS, DSS and PFS curves of MYB and MYBL1 were also performed from TCGA dataset. PAAD patients with high MYB expression had no difference in OS (Figure S2A, p=0.44), DSS (Figure S2B, p=0.634) and PFS (Figure S2C, p=0.821) compare to low expression sample. Similarly, there was no difference of OS (Figure S3A, p=0.24), DSS (Figure S3B, p=0.336) and PFS (Figure S3C, p=0.141) in pancreatic cancer patients with high MYBL1 expression compared to the low expression group. Taken together, the results of these analyses indicate that only the MYBL2 gene in the MYB family is associated with survival in pancreatic cancer patients, and that MYBL2 can serve as a reliable independent prognostic factor for pancreatic cancer patients.

### Biological Function of MYBL2 in PAAD

**Figure 6:**
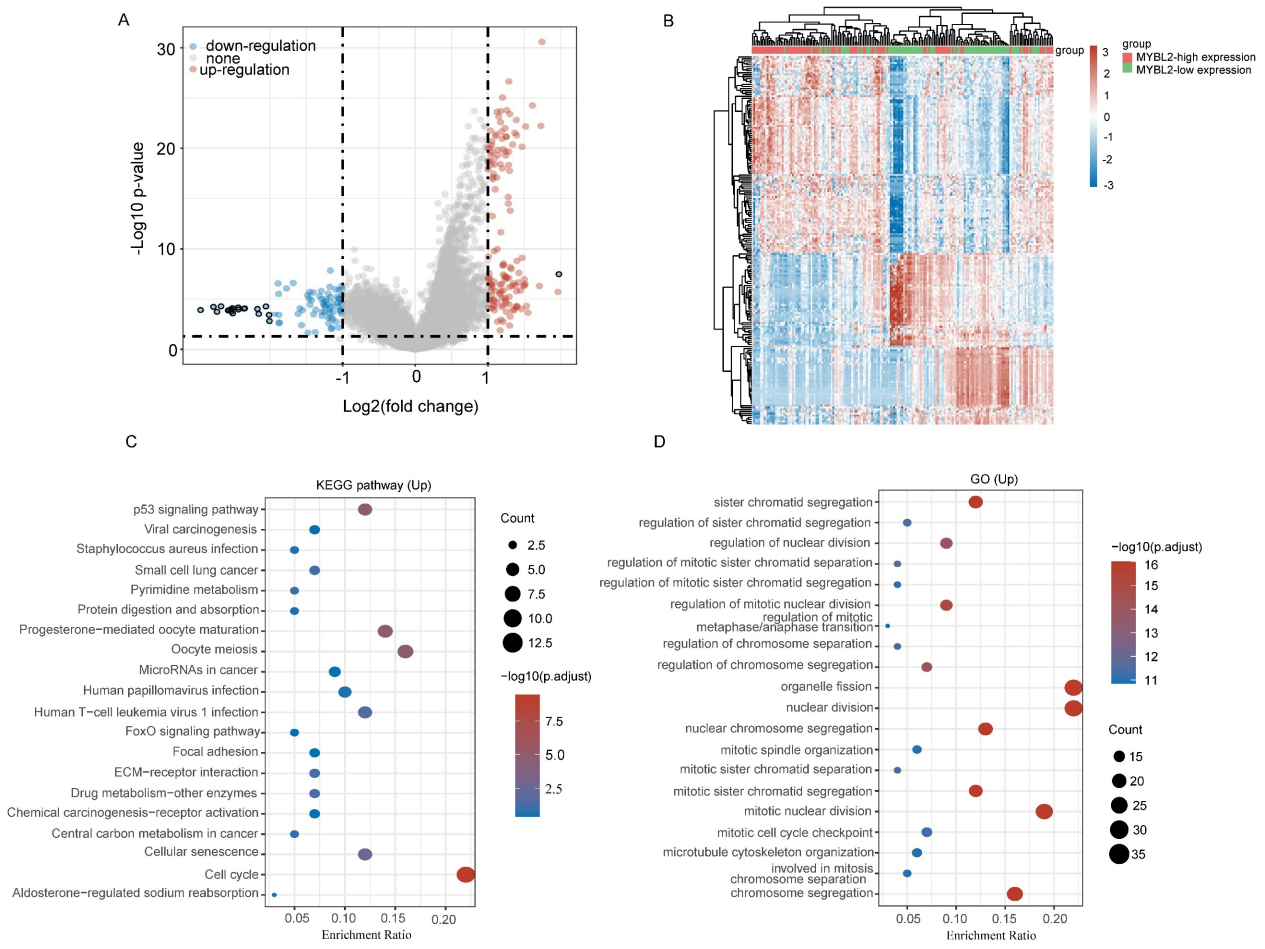
Functional inference of MYBL2 in PAAD. (A) Volcano plot analysis identifies DEGs. Red dots represent 139 upregulated genes; blue dots represent 87; grey dots indicate not significant. DEGs with the criteria set of |log2FC| > 1, adjusted p < 0.05. (B) Differential gene expression heat map with different colors representing trends in gene expression in pancreatic cancer tissues. (C) GO term enrichment results for differentially up-regulated genes. (D) Differential up-regulation of genes KEGG pathway enrichment results. Colors represent the significance of differential enrichment, the size of the circles represents the number of genes, the larger the circle, the greater the number of genes.

Next, to further confirm the potential biological function of MYBL2 in pancreatic cancer, we performed functional enrichment analysis on transcriptomic data from TCGA. we compared DEGs analysis between MYBL2-high group and MYBL2-low group (log2FC > 2 and adjusted P < 0.05). As shown in the volcano diagram, a total of 226 differentially expressed genes were screened including 139 up-regulated genes and 87 down-regulated genes (Figure 6A). Figure 6B shows the expression heatmap of differential genes, where different colors represent the expression trend in different tissues. This figure mainly shows the 50 up-regulated genes and 50 down-regulated genes with the largest differential changes (Figure 6B). To further determine the potential function of MYBL2, we performed GO annotation and KEGG enrichment analysis of up-regulated genes. The results of the analysis show that most of these up-regulated genes were mainly significantly enriched in cellular senescence, oocyte meiosis, p53 signaling pathway, cell cycle and pyrimidine metabolism, etc. (Figure 6C and D). To further confirm the role of MYBL2 in pancreatic cancer, the correlations between MYBL2 gene and pathway score was analysed with Spearman by using online software (https://www.aclbi.com/static/index.html).

**Figure 7:**
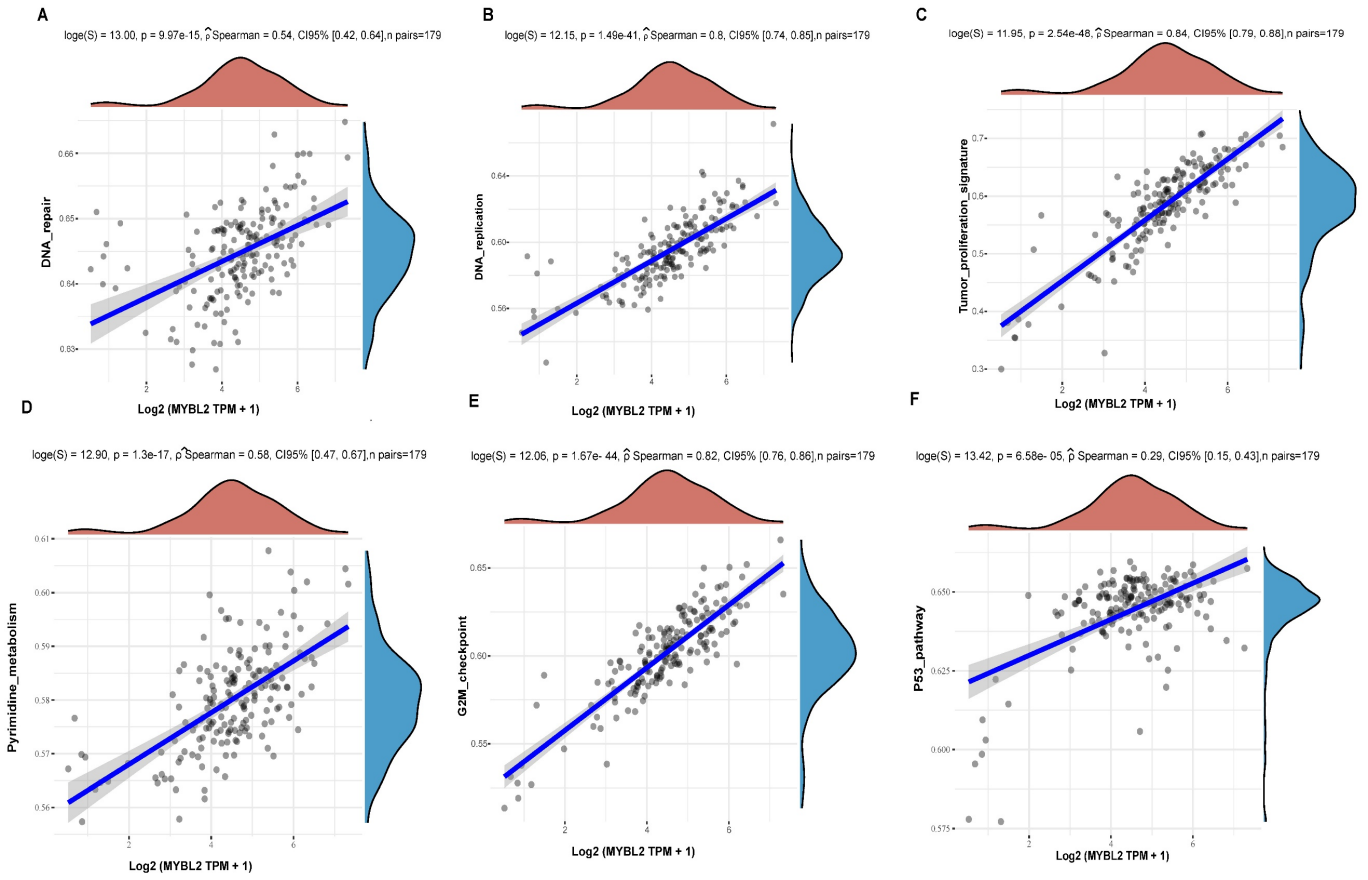
The correlations between MYBL2 gene and pathway score was analysed with Spearman. The abscissa represents the distribution of the gene expression, and the ordinate represents the distribution of the pathway score. (A)The correlations analysis between MYBL2 gene and DNA repair pathway. (B)The correlations analysis between MYBL2 gene and DNA replication pathway. (C) The correlations analysis between MYBL2 gene and tumor proliferation pathway. (D)The correlations analysis between MYBL2 gene and pyrimidine metabolism pathway. (E) The correlations analysis between MYBL2 gene and G2M checkpoint pathway. (F)The correlations analysis between MYBL2 gene and p53 pathway.

The results showed that MYBL2 expression in pancreatic cancer was significantly correlated with DNA repair (Figure 7A, p=9.97e-15), DNA replication (Figure 7B, p=1.49e-41), Tumor proliferation (Figure 7C, p=2.54e-48), Pyrimidine metabolism (Figure 7D, p=1.3e-17), G2M checkpoint (Figure 7E, p=1.67e-44) and p53 pathway (Figure 7F, p=6.58e-05). Comprehensive analysis shows that the impact of MYBL2 on tumor proliferation may be related to DNA replication, cell cycle, G2M checkpoint, p53, and pyrimidine metabolism pathways.

### The relationship between MYB family genes and TMB/MSI

**Figure 8:**
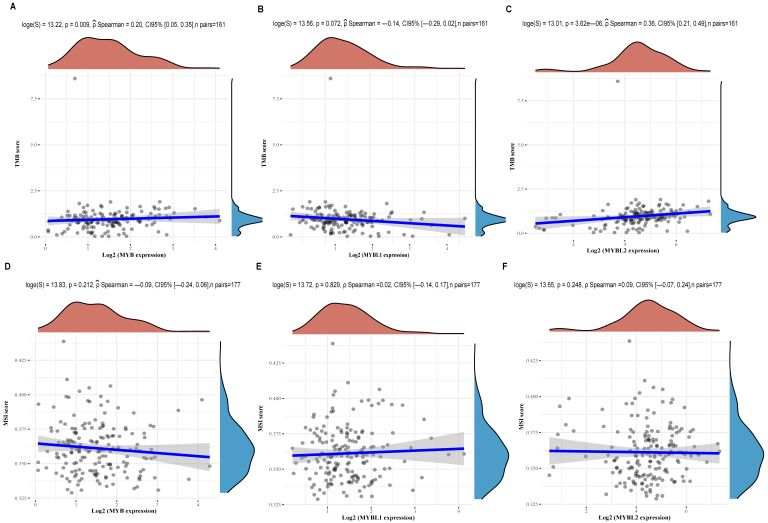
Correlation analysis between MYB family genes expression and TMB/MSI was performed using Spearmans method. (A) Relationship between TMB and MYB expression in pancreatic cancer. (B) Relationship between TMB and MYBL1 expression in pancreatic cancer. (C) Relationship between TMB and MYBL2 expression in pancreatic cancer. (D) Relationship between MSI and MYB expression in pancreatic cancer. (E) Relationship between MSI and MYBL1 expression in pancreatic cancer. (F) Relationship between MSI and MYBL2 expression in pancreatic cancer.

It is well recognized that TMB and MSI are biomarkers of immune checkpoint inhibitor (ICI) response. The present study, we performed a correlation analysis of MYB family genes and TMB/MSI expression using TCGA data. As shown in Figure 8A-C, the expression of MYB (Figure 8A, p=0.009) and MYBL2 (Figure 8C, p=3.62e-06) was significantly positively correlated with TMB in cancer, except for MYBL1(Figure 8B, p=0.072). Meanwhile, correlation analysis of MSI revealed no association between MYB (Figure 8D, p=0.212), MYBL1(Figure 8E, p=0.829) or MYBL2 (Figure 8F, p=0.248) expression and pancreatic cancer.

## Discussion

Pancreatic cancer remains one of the most aggressive malignancies affecting the digestive system, characterized by a grim prognosis and challenging early detection 20. Furthermore, there is a pressing need to identify novel prognostic indicators and therapeutic targets for pancreatic cancer, given the limited efficacy of current interventions such as surgery, radiotherapy, and immunotherapy in extending patient survival rates. The MYB family of genes encode oncogenic transcription factors that are crucial for regulating cell proliferation, differentiation, and autophagy in tumor cells, as well as for maintaining embryonic development and tissue homeostasis 21. Research on tumors has demonstrated that MYB family genes play a significant role in the advancement and growth of various types of tumors. For instance, MYB plays a crucial role in metabolic reprogramming to help pancreatic cancer cells survive in low oxygen conditions 22. Other research indicates that MYB may control the growth and genetic stability of pancreatic cancer cells by targeting various gene networks and signaling pathways, including EGFR and NF-κB 23. Specifically, a study has revealed that MYBL1 upregulates ANGPT2 expression, leading to angiogenesis and ultimately conferring resistance to sorafenib in hepatocellular carcinoma (HCC) cells. These findings suggest that MYBL1 could serve as a valuable diagnostic and therapeutic biomarker for individuals with HCC 24. In the field of oncology, the mammalian homologue of the oncoprotein v-Myb, known as c-Myb, has been linked to various human malignancies. Specifically, in studies focusing on leukemia, c-Myb has been correlated with a less favorable prognosis 25. A 2022 study found that circ-MYBL2 is significantly downregulated in pancreatic adenocarcinoma (PA) and exerts its suppressive effects on PA cells by downregulating miR-19a through methylation 26. The promotion of tumor growth by MYBL2 suggests its potential utility as a diagnostic and therapeutic target in non-small cell lung cancer, gastric cancer, castration-resistant prostate cancer, and endometrial carcinoma 27-30. Although many studies have confirmed the expression and roles of the MYB family in various cancer types and some have elucidated their underlying mechanisms, there has been no systematic comparative analysis of the expression and prognosis of MYB family members in pancreatic cancer. Therefore, in this study, we focused on the comparative analysis of the expression of MYB family genes and their impact on the prognosis of pancreatic cancer patients through various tumor-related databases, with the expectation of screening the most promising prognostic markers to guide the treatment of pancreatic cancer patients.

Initial findings suggest that pancreatic cancer patients exhibit significantly elevated levels of MYB, MYBL1, and MYBL2 gene expression at the RNA level compared to normal tissues, as indicated by analysis of data from the TCGA and TNMplot databases, consistent with previous research 11, 31. Subsequently, we proceeded to assess the protein expression levels of MYB, MYBL1, and MYBL2 in various types of cancer utilizing the HPA online analysis tool. The findings demonstrated that MYBL2 exhibited the highest expression levels in comparison to MYB and MYBL1, positioning it as the second highest among the tumors examined. Furthermore, our analysis revealed a positive correlation between MYBL2 expression and the pathologic grade and clinical stage of pancreatic cancer, suggesting a potential role of MYBL2 in influencing tumor progression. Research has demonstrated that MYBL2 overexpression serves as an independent prognostic factor in the progression of colorectal cancer 32. Our study identified that the expression of MYBL2, within the MYB family, is independently associated with overall survival (OS) in pancreatic cancer patients. Prognostic analysis indicated that MYBL2 is linked to unfavorable outcomes, including OS, disease-specific survival (DSS), and progression-free survival (PFS). These findings suggest that MYBL2 may function as an oncogene and serve as a prognostic biomarker for pancreatic cancer. Further analysis is needed to gain a more comprehensive understanding of the role of MYBL2 in different types of pancreatic cancer. Research on cancer-related genes has traditionally emphasized functional studies, with multiple studies highlighting the importance of MYBL2 in facilitating cell cycle progression 33. A DNA microarray analysis conducted on breast cancer samples revealed a significant correlation between MYBL2 expression and p53 mutations 34. Based on the findings of functional enrichment and correlation analysis in this study, it is evident that MYBL2 plays a significant role in tumor pathogenesis in pancreatic cancer, primarily through the modulation of DNA replication, the cell cycle G2M checkpoint, the p53 pathway, and pyrimidine metabolism. Immunotherapy has demonstrated efficacy as a treatment for cancer due to the ability of tumor cells to evade immune system surveillance, thereby facilitating unimpeded proliferation 35, 36. Considerable focus has been directed towards tumor mutation burden (TMB) within the realm of immunotherapy 37. Cancers characterized by high tumor mutational burden (TMB) or microsatellite instability (MSI) generally exhibit a more favorable prognosis and demonstrate a stronger correlation with positive responses to immunotherapy compared to low-TMB or microsatellite stable tumors 38. In this research, we utilized the online platform (https://www.aclbi.com/static/index.html) to examine the relationship between the expression of MYB family genes and the levels of TMB and MSI in pancreatic cancers sourced from the TCGA database. Our study revealed a significant positive correlation between MYBL2 expression and tumor mutational burden (TMB) in pancreatic cancer, while no such correlation was observed for MYB and MYBL1. These results indicate that MYBL2 may enhance the efficacy of immunotherapy in pancreatic cancer, whereas MYB and MYBL1 may not exhibit a similar sensitivity to this treatment modality.

## Conclusions

In summary, our comparative analysis of various databases revealed a strong correlation between MYBL2 expression within the MYB gene family and pancreatic cancer grading, staging, independent prognosis, and tumor mutational load (TMB). The findings suggest that MYBL2 may have a more pronounced impact on pancreatic cancer compared to MYB and MYBL1, particularly in its dual role as a prognostic marker and predictor of response to PD1 antibodies. This discovery offers a theoretical framework for conducting further comprehensive research on pancreatic cancer biomarkers. It is important to note that our conclusions are solely based on bioinformatics analysis. To confirm our results, it is imperative to delve deeper into the complex molecular pathways of MYBL2 in pancreatic cancer, in order to establish a robust theoretical basis for precise and personalized clinical interventions and management strategies for pancreatic cancer.

## Supplementary Material

Supplementary figures and tables.

## Figures and Tables

**Table 1:** Relationship between MYB expression level and clinicopathological variables and in pancreatic cancer patients

Clinical features	Characteristic	MYB high expression group	MYB low expression group	P_value
Status	Alive	42	44	
	Dead	48	45	0.825
Age	Mean (SD)	65.7 (11.8)	63.6 (9.9)	
	Median [MIN, MAX]	68 [35,85]	64 [39,88]	0.2
Gender	FEMALE	42	38	
	MALE	48	51	0.701
Race	ASIAN	6	5	
	BLACK	2	4	
	WHITE	80	78	0.678
new_tumor_event_type	Metastasis	29	29	
	Metastasis:Recurrence	2		
	Primary	1	1	
	Recurrence	9	11	0.928
Smoking	Non-smoking	27	39	
	Smoking	41	38	0.249
Radiation_therapy	Non-radiation	48	55	
	Radiation	14	18	0.936
History_of_neoadjuvant_treatment	No neoadjuvant	90	88	
	Yes, Radiation Prior to Resection		1	
Therapy_type	Ancillary:Chemotherapy	8	6	
	Chemotherapy	54	48	
	Chemotherapy:Vaccine	1		
	Chemotherapy:Hormone Therapy		1	0.992

* The absence of clinical data for certain samples resulted in a diminished sample size of cases exhibiting specific individual clinical characteristics, amounting to fewer than 179 instances.

**Table 2:** Relationship between MYBL1 expression level and clinicopathological variables and in pancreatic cancer patients

Clinical features	Characteristic	MYBL1 high expression group	MYBL1 low expression group	P_value
Status	Alive	42	44	
	Dead	48	45	0.825
Age	Mean (SD)	64 (10.6)	65.2 (11.3)	
	Median [MIN, MAX]	64 [40,88]	66 [35,85]	0.442
Gender	FEMALE	43	37	
	MALE	47	52	0.494
Race	ASIAN	2	9	
	BLACK	5	1	
	WHITE	81	77	0.027
new_tumor_event_type	Metastasis	34	24	
	Metastasis:Recurrence	2		
	Recurrence	9	11	
	Primary		2	0.426
Smoking	Non-smoking	32	34	
	Smoking	37	42	0.975
Radiation_therapy	Non-radiation	50	53	
	Radiation	15	17	1
History_of_neoadjuvant_treatment	No neoadjuvant	90	88	
	Yes, Radiation Prior to Resection		1	
Therapy_type	Ancillary:Chemotherapy	9	5	
	Chemotherapy	51	51	
	Chemotherapy:Hormone Therapy		1	
	Chemotherapy:Vaccine		1	0.473

* The absence of clinical data for certain samples resulted in a diminished sample size of cases exhibiting specific individual clinical characteristics, amounting to fewer than 179 instances.

**Table 3:** Relationship between MYBL2 expression level and clinicopathological variables and in pancreatic cancer patients

Clinical features	Characteristic	MYBL2 high expression group	MYBL2 low expression group	P_value
Status	Alive	34	52	
	Dead	56	37	0.009
Age	Mean (SD)	64.6 (11.3)	64.6 (10.5)	
	Median [MIN, MAX]	66 [35,84]	65 [39,88]	0.975
Gender	FEMALE	37	43	
	MALE	53	46	0.413
Race	ASIAN	7	4	
	BLACK	2	4	
	WHITE	80	78	0.482
new_tumor_event_type	Metastasis	36	22	
	Metastasis: Recurrence	1	1	
	Recurrence	9	11	
	Primary		2	0.402
Smoking	Non-smoking	36	30	
	Smoking	36	43	0.363
Radiation_therapy	Non-radiation	55	48	
	Radiation	14	18	0.453
History_of_neoadjuvant_treatment	No neoadjuvant	90	88	
	Yes, Radiation Prior to Resection		1	
Therapy_type	Ancillary: Chemotherapy	6	8	
	Chemotherapy	51	51	
	Chemotherapy: Hormone Therapy		1	
	Chemotherapy: Vaccine		1	0.829

* The absence of clinical data for certain samples resulted in a diminished sample size of cases exhibiting specific individual clinical characteristics, amounting to fewer than 179 instances.

## Abbreviations

TWIST1: twist family bHLH transcription factor 1
TMB: tumor mutational burden
MSI: microsatellite instability
PD1: programmed cell death protein 1
OS: Overall Survival
DSS: disease-specific survival
PFS: progression-free survival
ANGPT2: angiopoietin 2
EGFR: epidermal growth factor receptor
NF-κB: nuclear factor of kappa light polypeptide gene enhancer in B cells 1
ROC: Receiver Operating Characteristic
AUC: Area Under roc Curve
HPA: Human Protein Atls
p53: tumor protein 53
