# Supplementary material for: MYBL2 is a Novel Independent Prognostic Biomarker and Correlated with TMB in pancreatic cancer
Source: J Cancer. 2024 Jun 11;15(13):4360–73. doi: 10.7150/jca.96320 (PMC11212096; doi:10.7150/jca.96320)
Supplement: Supplementary file 1 — Supplementary figures and tables. [file jcav15p4360s1.pdf]

Supplementary Figure S1 The correlation between MYB family genes and the stage,grade of pancreatic cancer. (A) MYB gene violin plots of patient pathological stage by GEPIA.(B) MYBL1 gene violin plots of patient pathological stage by GEPIA.(C) MYBL2 gene violin plots of patient pathological stage by GEPIA.(D) Expression of MYB in PAAD based on tumor grade by UALCANplatform.(E) Expression of MYBL1 in PAAD based on tumor grade by UALCAN platform.(F) Expression of MYBL2 in PAAD based on tumor grade by UALCAN platform.

Supplementary Figure S2 The correlation between MYB expression and OS (A), DSS (B) and PFS (C) from TCGA dataset, comparison among different groups was made by log-rank test. The time ROC(v 0.4) analysis was used to compare the predictive accuracy of MYB gene for overall survival (OS)(D), (DSS)(E),(PFS)(F),respectively.

Supplementary Figure S3 The correlation between MYBL1 expression and OS (A), DSS (B) and PFS (C) from TCGA dataset, comparison among different groups was made by log-rank test. The time ROC(v 0.4) analysis was used to compare the predictive accuracy of MYBL1 gene for overall survival (OS)(D), (DSS)(E),(PFS)(F), respectively.

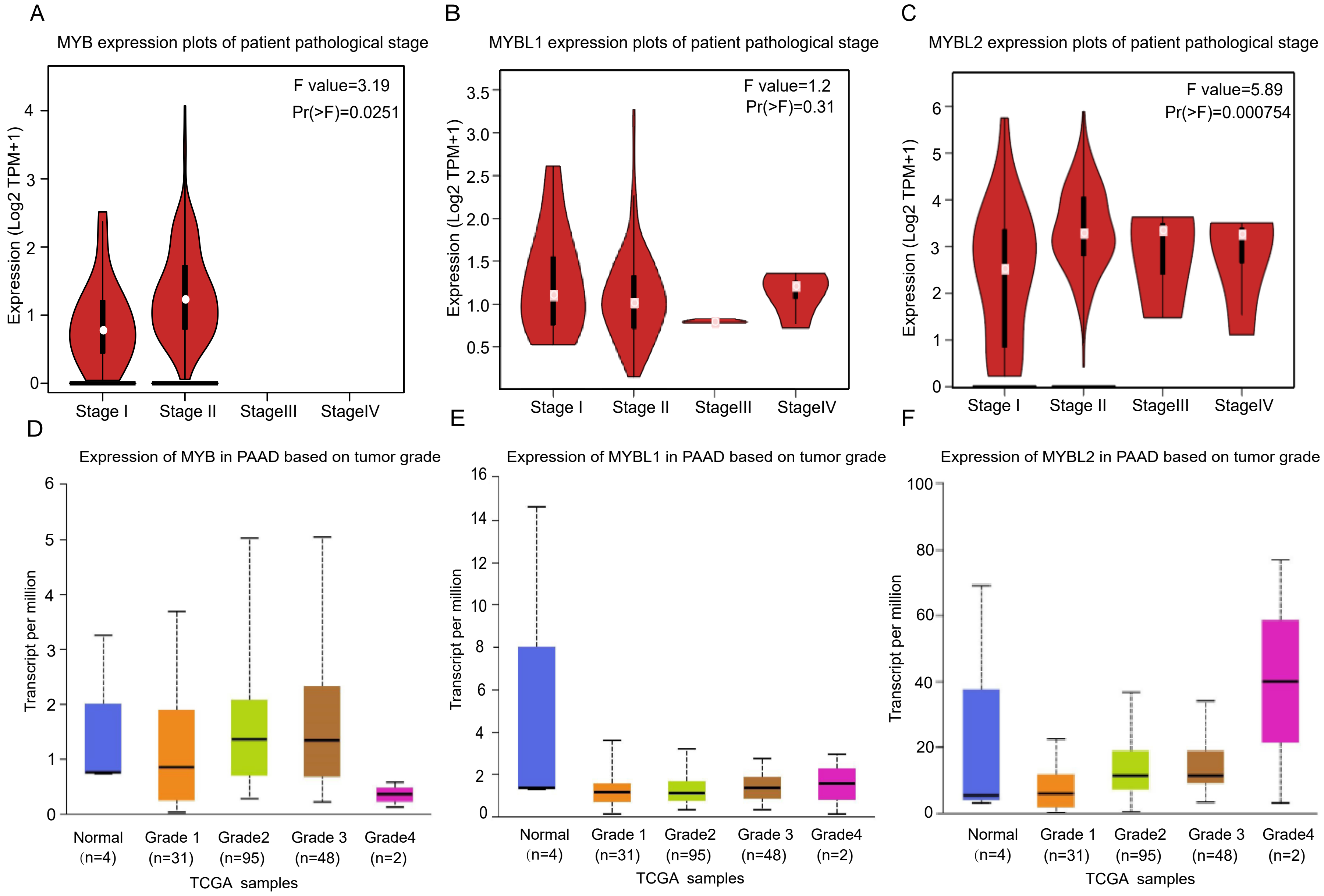
A
MYB expression plots of patient pathological stage
F value=3.19
Pr(>F)=0.0251
Expression (Log2 TPM+1)
Stage I
Stage II
StageIII
StageIV
B
MYBL1 expression plots of patient pathological stage
F value=1.2
Pr(>F)=0.31
Expression (Log2 TPM+1)
Stage I
Stage II
StageIII
StageIV
C
MYBL2 expression plots of patient pathological stage
F value=5.89
Pr(>F)=0.000754
Expression (Log2 TPM+1)
Stage I
Stage II
StageIII
StageIV
D
Expression of MYB in PAAD based on tumor grade
Transcript per million
Normal (n=4)
Grade 1 (n=31)
Grade2 (n=95)
Grade 3 (n=48)
Grade4 (n=2)
TCGA samples
E
Expression of MYBL1 in PAAD based on tumor grade
Transcript per million
Normal (n=4)
Grade 1 (n=31)
Grade2 (n=95)
Grade 3 (n=48)
Grade4 (n=2)
TCGA samples
F
Expression of MYBL2 in PAAD based on tumor grade
Transcript per million
Normal (n=4)
Grade 1 (n=31)
Grade2 (n=95)
Grade 3 (n=48)
Grade4 (n=2)
TCGA samples

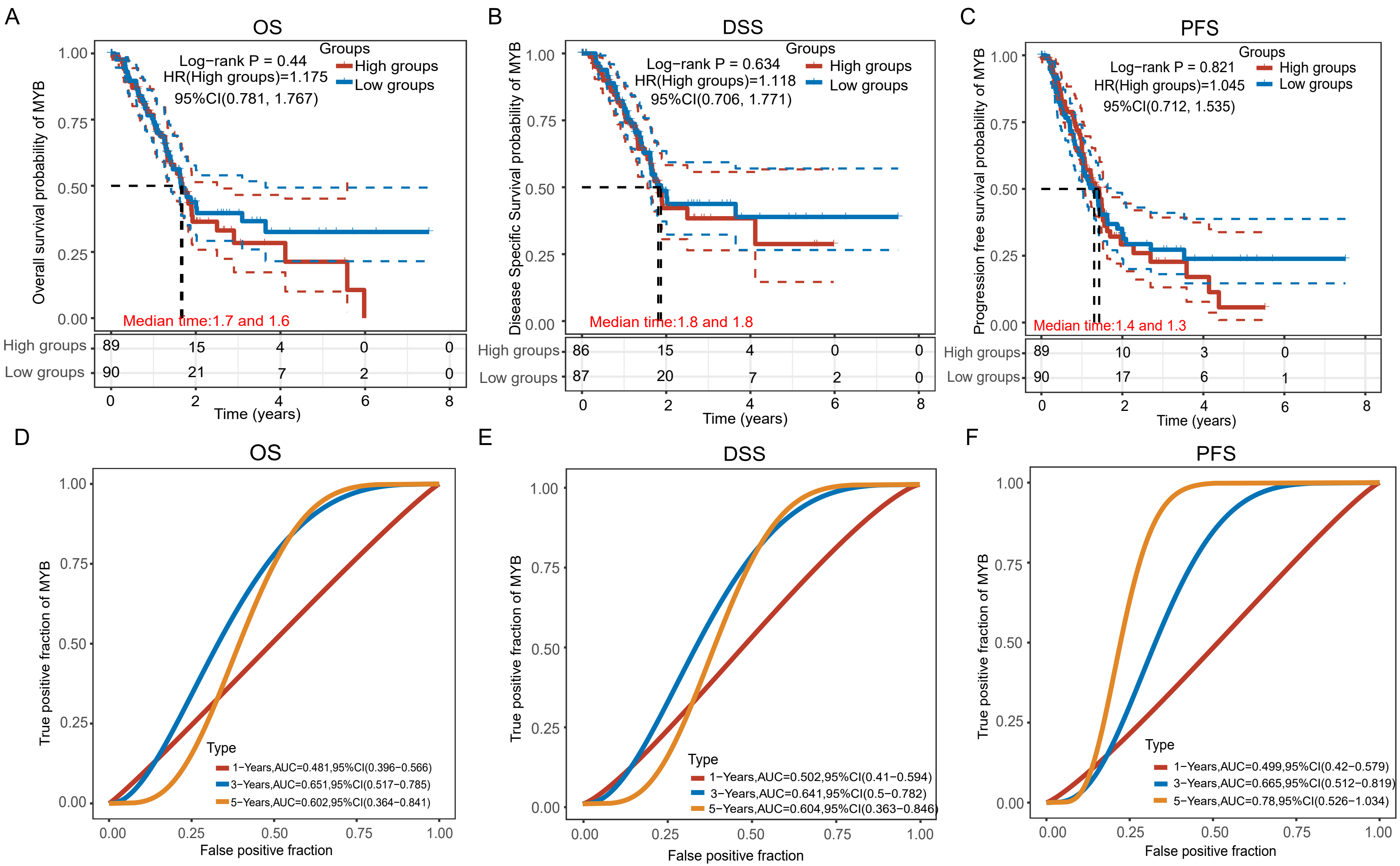

A
OS
Log−rank P = 0.44
HR(High groups)=1.175
95%CI(0.781, 1.767)
Groups
High groups
Low groups
Overall survival probability of MYB
Median time:1.7 and 1.6
High groups 89 15 4 0 0
Low groups 90 21 7 2 0
Time (years)
B
DSS
Log−rank P = 0.634
HR(High groups)=1.118
95%CI(0.706, 1.771)
Groups
High groups
Low groups
Disease Specific Survival probability of MYB
Median time:1.8 and 1.8
High groups 86 15 4 0 0
Low groups 87 20 7 2 0
Time (years)
C
PFS
Log−rank P = 0.821
HR(High groups)=1.045
95%CI(0.712, 1.535)
Groups
High groups
Low groups
Progression free survival probability of MYB
Median time:1.4 and 1.3
High groups 89 10 3 0
Low groups 90 17 6 1
Time (years)
D
OS
True positive fraction of MYB
Type
1−Years,AUC=0.481,95%CI(0.396−0.566)
3−Years,AUC=0.651,95%CI(0.517−0.785)
5−Years,AUC=0.602,95%CI(0.364−0.841)
False positive fraction
E
DSS
True positive fraction of MYB
Type
1−Years,AUC=0.502,95%CI(0.41−0.594)
3−Years,AUC=0.641,95%CI(0.5−0.782)
5−Years,AUC=0.604,95%CI(0.363−0.846)
False positive fraction
F
PFS
True positive fraction of MYB
Type
1−Years,AUC=0.499,95%CI(0.42−0.579)
3−Years,AUC=0.665,95%CI(0.512−0.819)
5−Years,AUC=0.78,95%CI(0.526−1.034)
False positive fraction

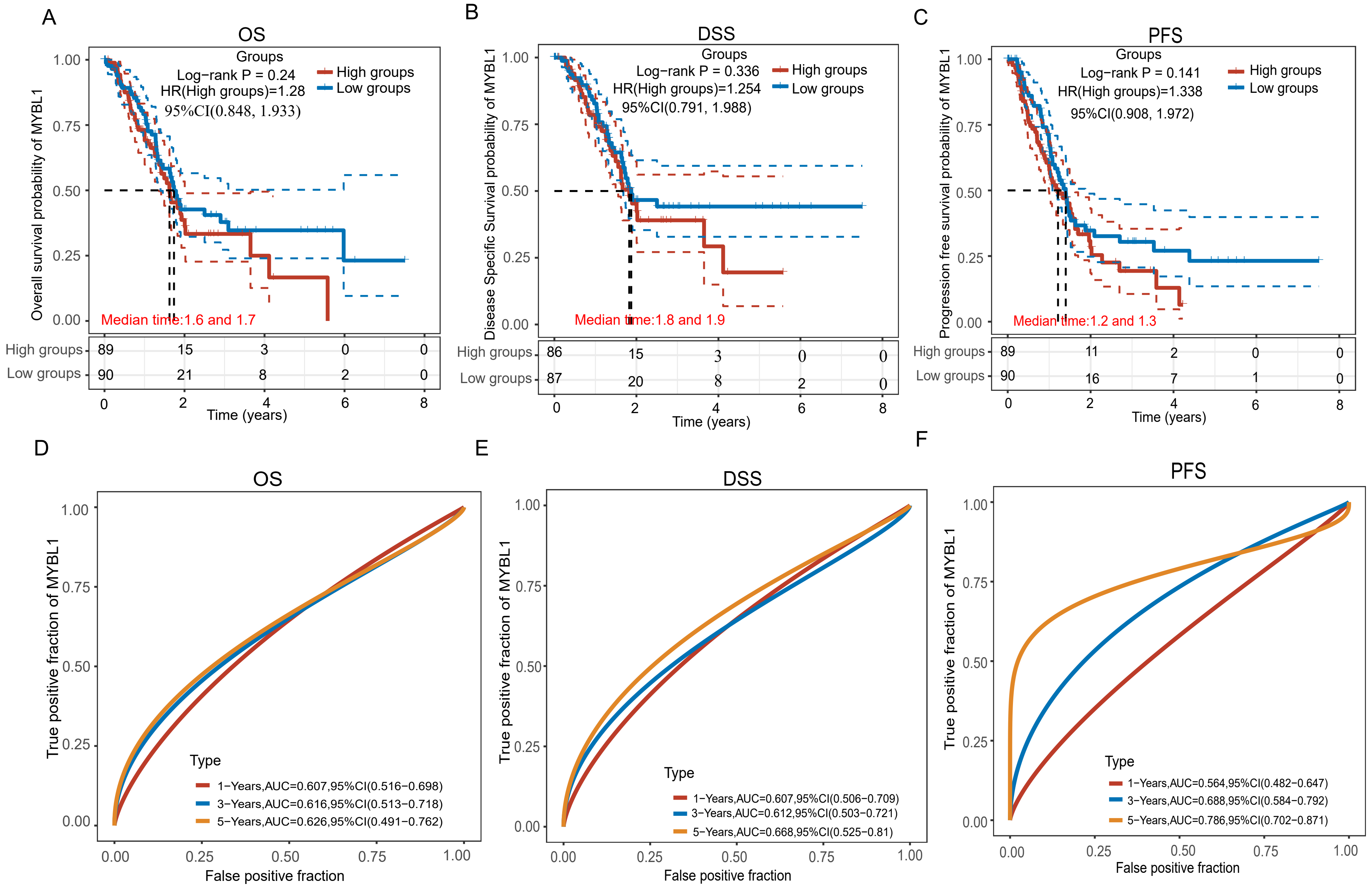

A
OS
Groups
Log−rank P = 0.24
HR(High groups)=1.28
95%CI(0.848, 1.933)
High groups
Low groups
Overall survival probability of MYBL1
Median time:1.6 and 1.7
High groups 89 15 3 0 0
Low groups 90 21 8 2 0
Time (years)
B
DSS
Groups
Log−rank P = 0.336
HR(High groups)=1.254
95%CI(0.791, 1.988)
High groups
Low groups
Disease Specific Survival probability of MYBL1
Median time:1.8 and 1.9
High groups 86 15 3 0 0
Low groups 87 20 8 2 0
Time (years)
C
PFS
Groups
Log−rank P = 0.141
HR(High groups)=1.338
95%CI(0.908, 1.972)
High groups
Low groups
Progression free survival probability of MYBL1
Median time:1.2 and 1.3
High groups 89 11 2 0 0
Low groups 90 16 7 1 0
Time (years)
D
OS
True positive fraction of MYBL1
False positive fraction
Type
1−Years,AUC=0.607,95%CI(0.516−0.698)
3−Years,AUC=0.616,95%CI(0.513−0.718)
5−Years,AUC=0.626,95%CI(0.491−0.762)
E
DSS
True positive fraction of MYBL1
False positive fraction
Type
1−Years,AUC=0.607,95%CI(0.506−0.709)
3−Years,AUC=0.612,95%CI(0.503−0.721)
5−Years,AUC=0.668,95%CI(0.525−0.81)
F
PFS
True positive fraction of MYBL1
False positive fraction
Type
1−Years,AUC=0.564,95%CI(0.482−0.647)
3−Years,AUC=0.688,95%CI(0.584−0.792)
5−Years,AUC=0.786,95%CI(0.702−0.871)
